# Bilateral Ultrasonographic Mapping and Morphometry of the Infraclavicular Brachial Plexus: A Prospective Multicenter Study

**DOI:** 10.3390/jcm15176892

**Published:** 2026-09-06

**Authors:** Petar-Preslav Petrov, Delyan Dimitrov, Darina Barbutska, Tanya Kitova, Antoaneta Fasova, Pavel Timonov, Yasen Getsov, Andreas Chatzigiannis, Nikoleta Dimitrova, Ivaylo Minev

**Affiliations:** 1 Department of Anatomy, Histology and Embryology, Faculty of Medicine, Medical University of Plovdiv, 4002 Plovdiv, Bulgaria; 2 Faculty of Medicine, Medical University of Plovdiv, 4002 Plovdiv, Bulgaria; 3 Department of Forensic Medicine and Deontology, Faculty of Medicine, Medical University of Plovdiv, 4002 Plovdiv, Bulgaria; 4 Department of Anesthesiology and Intensive Care, Acibadem City Clinic University Hospital Vitosha, 1415 Sofia, Bulgaria; 5 Clinic of Anesthesiology and Intensive Care, University Multiprofile Hospital for Active Treatment “St. George”, 4000 Plovdiv, Bulgaria; 6 Department of Occupational Diseases including Clinical Allergology Activities, University Multiprofile Hospital for Active Treatment “St. George”, 4002 Plovdiv, Bulgaria; 7 Department of Occupational Diseases, Clinical Allergology and Toxicology, Faculty of Medicine, Medical University of Plovdiv, 4002 Plovdiv, Bulgaria; 8 Department of Anesthesiology and Intensive Care Medicine, Faculty of Medicine, Medical University of Plovdiv, 4002 Plovdiv, Bulgaria

**Keywords:** brachial plexus cords, infraclavicular region, axillary artery, axillary vein, ultrasound anatomy, infraclavicular block, regional anesthesia

## Abstract

**Background/Objectives:** The infraclavicular region contains a complex arrangement of the brachial plexus cords and axillary vessels. Accurate ultrasonographic characterization of these relationships is important for anatomical image interpretation and has direct relevance to ultrasound-guided infraclavicular procedures. This study aimed to evaluate the bilateral topographic distribution and morphometric relationships of the lateral, medial, and posterior cords relative to the axillary artery and axillary vein. **Methods:** This prospective, multicenter, observational anatomical–ultrasonographic study included 50 adults (25 women and 25 men), each examined bilaterally, yielding 100 potential side-specific examinations. The lateral cord (LC), medial cord (MC), posterior cord (PC), and axillary vein (AV) were mapped using a 12-sector radial model centered on the axillary artery (AA). Center-to-center intercord, cord-to-AA, and cord-to-AV distances were measured. Topographic confidence intervals were estimated using participant-level cluster bootstrap resampling, and bilateral morphometric data were analyzed using linear mixed-effects models. **Results:** The LC was located in sectors 10–11 in 85/94 examinations (90.4%; 95% CI, 84.4–95.7%), the MC in sectors 2–4 in 84/89 (94.4%; 95% CI, 89.2–98.9%), the PC in sectors 6–7 in 78/83 (94.0%; 95% CI, 88.6–98.8%), and the AV in sectors 4–5 in 95/100 (95.0%; 95% CI, 90.0–99.0%). Mean intercord distances were 11.20 ± 1.18 mm for LC/MC, 7.30 ± 1.61 mm for LC/PC, and 5.70 ± 1.10 mm for MC/PC. Mean cord-to-AA distances were 7.60 ± 0.74, 4.60 ± 0.93, and 6.60 ± 0.91 mm for the LC, MC, and PC, respectively. Mean cord-to-AV distances were 15.50 ± 2.01, 6.60 ± 1.22, and 8.50 ± 1.10 mm, respectively. Mixed-effects models demonstrated significant differences among measurements within each distance family (all *p* < 0.001), without significant overall side or sex effects. **Conclusions:** The brachial plexus cords and AV demonstrated a consistent topographic organization around the AA during bilateral mid-infraclavicular ultrasonographic assessment. The MC showed the shortest center-to-center distance to both the AA and AV, and the three cords formed a non-equidistant spatial configuration.

## 1. Introduction

The infraclavicular region is a clinically important anatomical area containing the cords of the brachial plexus in close relationship with the axillary vessels. At this level, the lateral, medial, and posterior cords are arranged around the axillary artery, which serves as a principal ultrasonographic landmark for anatomical orientation. High-resolution ultrasonography enables detailed visualization of the supra- and infraclavicular components of the brachial plexus and their relationships with surrounding structures [1].

The introduction of ultrasound guidance has substantially changed peripheral nerve blockade by enabling real-time visualization of neural structures, adjacent vessels, needle advancement, and local anesthetic spread. Systematic reviews and meta-analyses have demonstrated advantages of ultrasound guidance compared with electrical neurostimulation or landmark-based techniques [2,3]. Ultrasound guidance has also been associated with shorter block performance times, fewer needle passes, faster onset, and the possibility of reducing local anesthetic requirements [4,5]. Beyond efficacy, ultrasound guidance has important safety implications and may reduce inadvertent vascular puncture and the risk of local anesthetic systemic toxicity [6,7]. Nevertheless, ultrasound does not eliminate procedural complications, and safe performance remains dependent on accurate image interpretation and detailed knowledge of regional sonoanatomy [8].

The infraclavicular approach is particularly dependent on accurate recognition of the spatial relationships between the brachial plexus cords and the axillary vessels. Previous clinical and review data support infraclavicular brachial plexus block for anesthesia and analgesia of the distal upper limb, and early ultrasound-guided studies demonstrated the value of direct visualization for identifying the plexus and adjacent vascular structures [9,10]. A systematic review of ultrasound-guided methods for brachial plexus blockade has also emphasized the diversity of approaches and techniques used in the region [11].

Kumar et al. characterized the topographic sonoanatomy of the infraclavicular brachial plexus using a 12-sector radial model centered on the axillary artery and quantified center-to-center distances between the individual cords and the artery [12]. Their study provided an important framework for describing the relative positions of the lateral, medial, and posterior cords and the axillary vein. However, quantitative relationships among the three cords themselves and individual center-to-center distances from each cord to the axillary vein were not evaluated. Furthermore, the examination was unilateral, preventing within-participant assessment of right–left variation.

The present study was designed as a prospective multicenter replication and extension of the 12-sector ultrasonographic mapping framework described by Kumar et al. The primary aim was to assess whether the previously reported artery-centered topographic distribution of the lateral, medial, and posterior cords and the axillary vein could be reproduced in an independent cohort using a standardized infraclavicular ultrasound protocol. The study further extended the previous methodology by performing bilateral examinations and by quantifying intercord distances and individual center-to-center distances from each cord to the axillary vein. Secondary analyses assessed right–left differences in sector distribution and side- and sex-related effects on morphometric measurements.

## 2. Materials and Methods

### 2.1. Study Design and Ethics

This was a prospective, multicenter, observational anatomical–ultrasonographic study of the infraclavicular region and its anatomical structures. The study was conducted at Acibadem City Clinic UMHAT Vitosha, Sofia, Bulgaria, and University Multiprofile Hospital for Active Treatment “St. George”, Plovdiv, Bulgaria. Recruitment and examinations were performed between May and June 2026.

The study was approved by the Ethics Committee of the Medical University of Plovdiv, Bulgaria (Approval Code R-KNE-65/08.05.2026; Protocol No. 5/29.04.2026). The study was conducted in accordance with the Declaration of Helsinki. Written informed consent was obtained from all participants prior to inclusion in the study.

### 2.2. Participants

Fifty adult participants aged between 20 and 60 years were included in the study. The study population consisted of healthy volunteers and hospitalized patients who underwent ultrasonographic evaluation of the infraclavicular region. During the recruitment period, 50 individuals were assessed for eligibility. All 50 met the eligibility criteria, provided written informed consent, and were enrolled in the study. No individuals were excluded before enrollment, and no enrolled participants withdrew or were excluded subsequently. Thirty participants were recruited at the Plovdiv center and 20 at the Sofia center; the study population comprised 30 healthy volunteers and 20 hospitalized patients.

The study population included 25 male and 25 female participants. Equal representation of men and women was prespecified before recruitment to allow balanced descriptive assessment according to sex. Both infraclavicular regions were examined in every participant, resulting in 50 right-sided and 50 left-sided potential examinations.

Failure to visualize an individual cord with sufficient confidence was not considered a reason for excluding the participant. No participant was excluded because one or more brachial plexus cords could not be visualized with sufficient confidence.

Age, height, body weight, and body mass index (BMI) were recorded for each participant.

The target sample of 50 participants was prespecified on the basis of recruitment feasibility. Because the primary objectives were descriptive and morphometric rather than hypothesis-driven, no formal a priori hypothesis-testing power calculation was performed. To characterize the precision afforded by this sample, 50 independent participants provided an approximate two-sided 95% confidence-interval half-width of 13.9 percentage points for a proportion of 0.50 under the conservative binomial assumption, and approximately 8.3 percentage points for a proportion of 0.90. Bilateral examination increased the number of side-specific anatomical observations but was not considered to double the number of independent participants; analyses of bilateral data therefore accounted for within-participant dependence.

### 2.3. Ultrasound Equipment and Settings

Ultrasonographic examinations were performed using one of three ultrasound systems available at the participating centers: Venue™ (GE HealthCare, Chicago, IL, USA), LOGIQ™ e (GE HealthCare, Chicago, IL, USA), or Affiniti 70 (Philips Ultrasound LLC, Bothell, WA, USA). All systems were equipped with high-frequency linear transducers suitable for imaging superficial neural and vascular structures in B-mode.

Gain, depth, focus, and other imaging parameters were individually optimized for each participant to obtain clear visualization of the neural and vascular structures in the infraclavicular region. When necessary, vascular structures were additionally confirmed based on their pulsatility, compressibility, and anatomical location. Color Doppler was used when additional confirmation of vascular structures was required.

### 2.4. Participant and Probe Positioning

All examinations were performed under standardized conditions in a controlled temperature environment. Each participant was placed in the supine position, with the examined arm abducted at 90° relative to the body, the elbow flexed at 90°, and the head rotated to the contralateral side.

**Figure 1:**
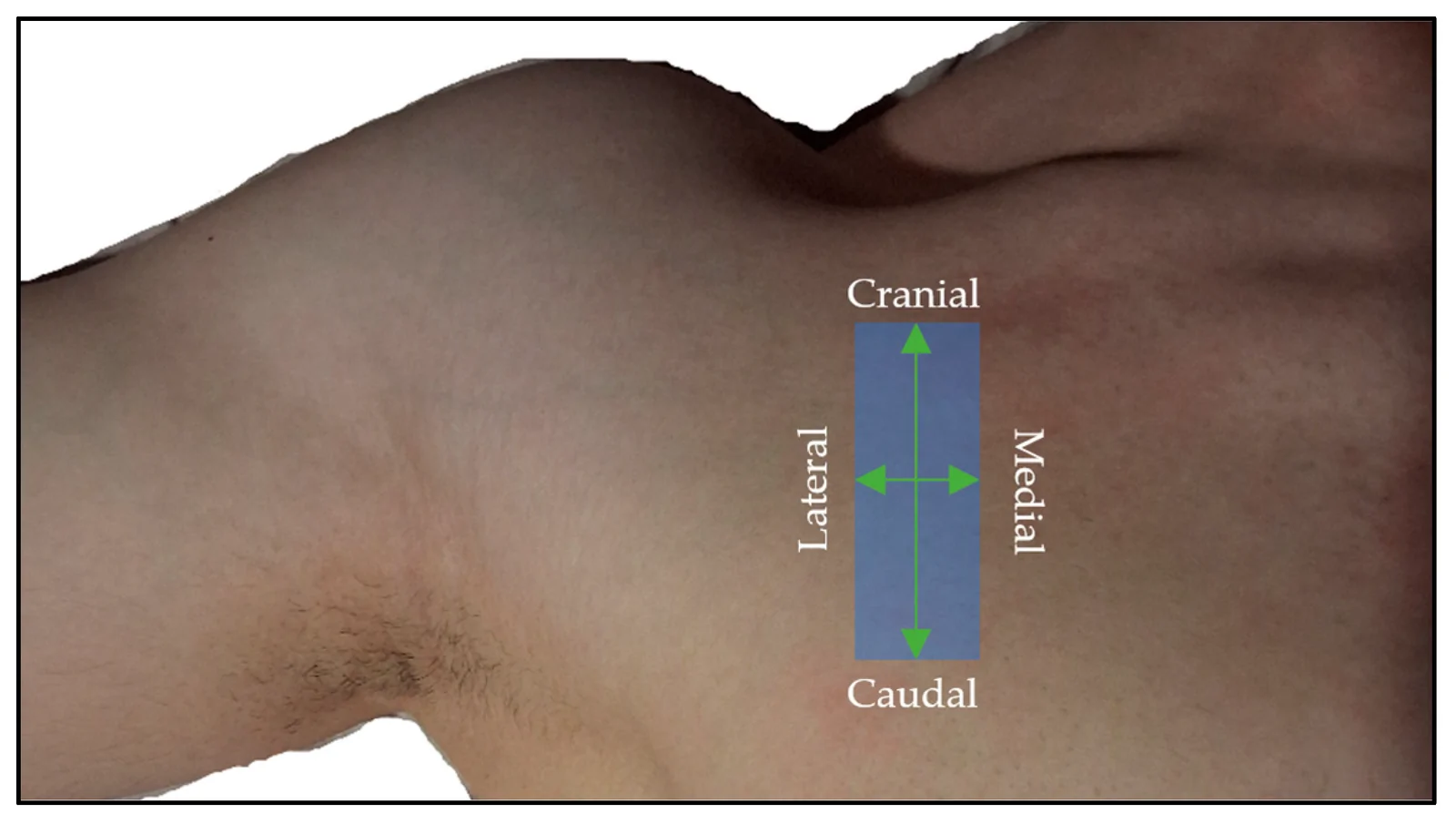
Transducer position for infraclavicular ultrasonographic examination. The linear transducer was positioned in the mid-infraclavicular region, approximately beneath the middle portion of the clavicle, in a parasagittal craniocaudal orientation. The blue rectangle indicates the approximate transducer position, and the arrows indicate the cranial, caudal, medial, and lateral directions.

Ultrasound gel was applied to the infraclavicular region. The linear ultrasound transducer was positioned in the mid-infraclavicular region, approximately beneath the middle portion of the clavicle, in a parasagittal orientation, with its long axis directed craniocaudally and approximately perpendicular to the clavicle (Figure 1). The transducer was adjusted to obtain a short-axis view of the axillary artery, axillary vein, and surrounding brachial plexus cords. Minimal transducer pressure was applied whenever possible to reduce compression-related displacement or deformation of the axillary vein.

### 2.5. Ultrasonographic Anatomy

The pectoralis major and pectoralis minor muscles, the clavipectoral fascia, the axillary artery, the axillary vein, and the three cords of the brachial plexus were identified in the infraclavicular region.

The axillary artery was used as the main anatomical landmark for orientation. It was identified as a pulsatile, predominantly circular vascular structure with hyperechoic walls and an anechoic lumen. The axillary vein was identified as a non-pulsatile and compressible vascular structure with variable morphology.

The lateral, medial, and posterior cords of the brachial plexus were identified around the axillary artery according to their anatomical relationship with the vessel. The lateral cord was typically located lateral or superolateral to the axillary artery, the medial cord medial or anteromedial to the artery, and the posterior cord posterior or deep to the artery. The cords were visualized as oval or rounded neural structures with variable echogenicity, usually showing a hypoechoic fascicular pattern surrounded by hyperechoic connective tissue.

### 2.6. Measurement Protocol

All ultrasound examinations were performed in real time and documented using frozen-frame images. Measurements were obtained using the built-in caliper tools of the ultrasound system. For each side, measurements were performed on a selected frozen image providing the clearest visualization of the relevant anatomical structures.

The following distances were measured:

- Intercord distances, including lateral cord to medial cord (LC/MC), lateral cord to posterior cord (LC/PC), and medial cord to posterior cord (MC/PC);
- Distances from the cords to the axillary artery, including lateral cord to axillary artery (LC/AA), medial cord to axillary artery (MC/AA), and posterior cord to axillary artery (PC/AA);
- Distances from the cords to the axillary vein, including lateral cord to axillary vein (LC/AV), medial cord to axillary vein (MC/AV), and posterior cord to axillary vein (PC/AV).

**Figure 2:**
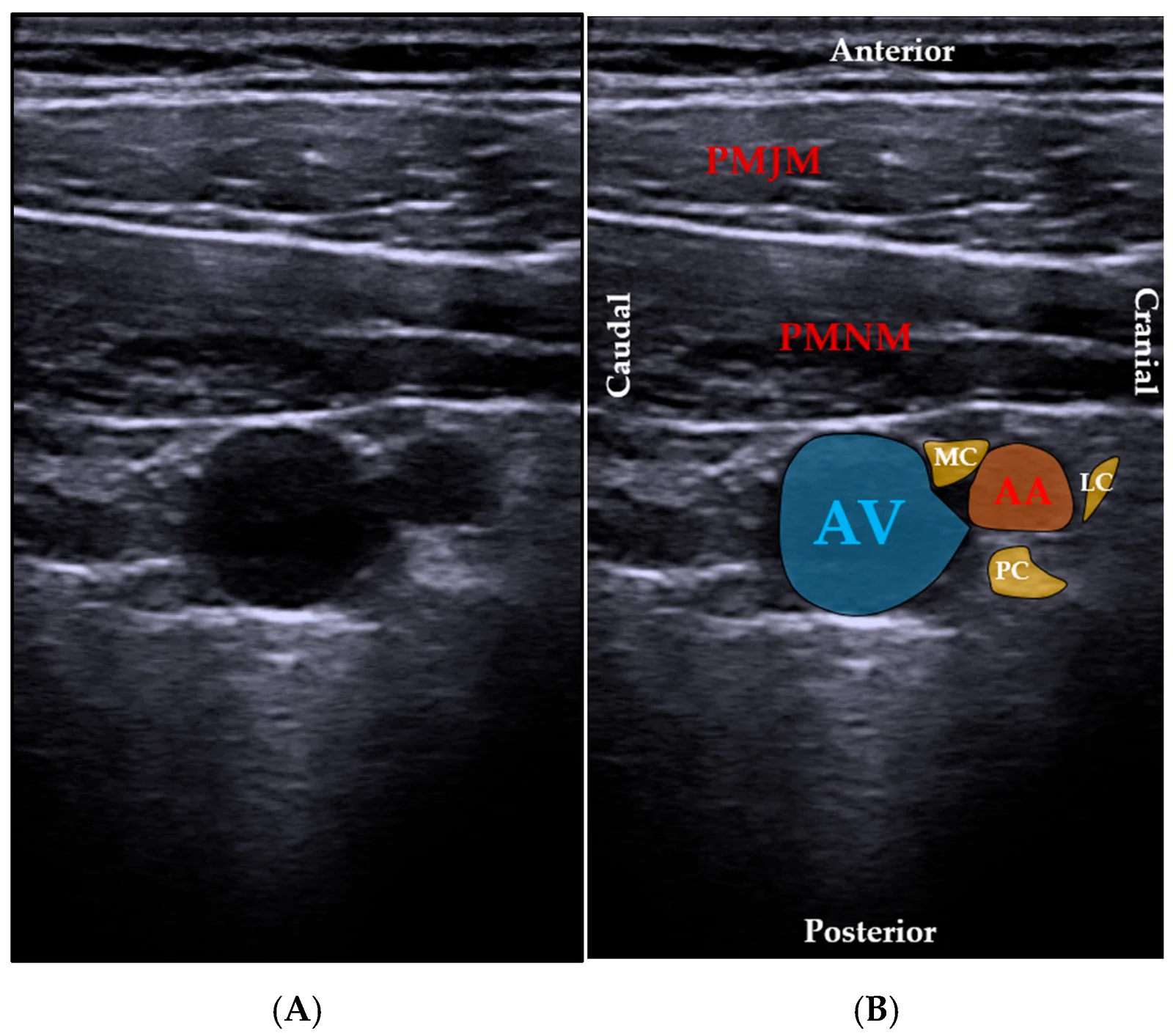
Ultrasound images of the brachial plexus cords, axillary artery and axillary vein, and the pectoralis major and pectoralis minor muscles in the infraclavicular region. (**A**). Unannotated ultrasound image of the infraclavicular region. (**B**). Ultrasound image of the infraclavicular region with the identified anatomical elements. Legend: PMJM—Pectoralis major muscle, PMNM—pectoralis minor muscle, AA—Axillary artery, AV—Axillary vein, LC—Lateral cord, MC—Medial Cord, PC—Posterior Cord.

Anatomical elements of the infraclavicular region are presented in Figure 2.

All distances were measured from the center of one structure to the center of the corresponding structure. Intercord distances were measured from the center of one cord to the center of the corresponding cord. The distances from each cord to the axillary artery and axillary vein were measured from the center of the cord to the center of the corresponding vascular structure. All measurements were expressed in millimeters. The center of each structure was defined visually as the midpoint of its sonographic cross-sectional profile on the selected frozen-frame image.

All morphometric measurements used in the primary analysis were obtained by the primary operator.

When an individual cord could not be visualized with sufficient confidence for reliable identification and center-point placement, the corresponding structure-specific measurement was recorded as missing. The participant and the remaining adequately visualized structures from the same examination were retained in the analysis. Missing values were not imputed.

**Figure 3:**
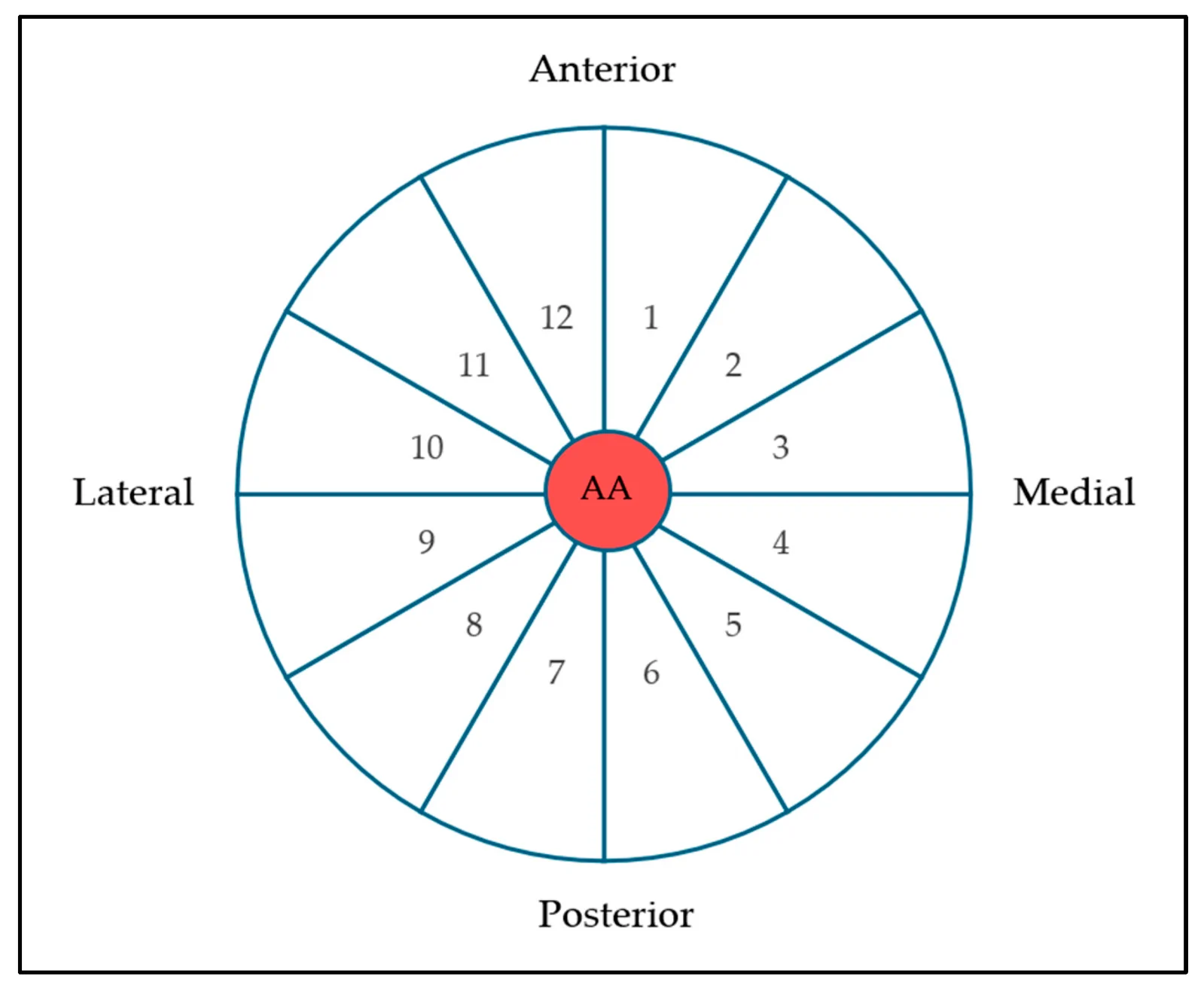
Twelve-sector radial model used for topographic assessment of the lateral, medial, and posterior cords and the axillary vein relative to the axillary artery, based on the mapping framework described by Kumar et al. [12].

In addition to the morphometric measurements, the topographic relationships of the lateral, medial, and posterior cords of the brachial plexus, as well as the axillary vein, were assessed in relation to the axillary artery. The 12-sector radial mapping framework described by Kumar et al. [12] was used, with the axillary artery positioned at the center as the main ultrasonographic landmark (Figure 3). Each cord and the axillary vein were assigned to the corresponding sector according to the position of their center relative to the center of the axillary artery on the selected parasagittal infraclavicular ultrasound image, with the axillary artery visualized in short axis. If a cord could not be identified with sufficient confidence, no sector was assigned for that structure.

### 2.7. Statistical Analysis

Statistical analyses were performed using IBM SPSS Statistics version 19.0 (IBM Corp., Armonk, NY, USA), jamovi version 2.6.44 (The jamovi Project, Sydney, Australia), and Microsoft Excel 2016 (Microsoft Corporation, Redmond, WA, USA).

Continuous participant characteristics and morphometric variables were summarized using descriptive statistics, including mean and standard deviation (SD). For morphometric measurements, 95% confidence intervals for the mean, median with interquartile range (IQR), and minimum–maximum range were additionally reported. Categorical variables were summarized as counts and percentages. Morphometric analyses were performed using available observations without imputation of structure-specific missing values.

Topographic distributions of the lateral cord, medial cord, posterior cord, and axillary vein were summarized according to the 12-sector radial model using counts, structure-specific denominators (n/N), percentages, and 95% confidence intervals. Confidence intervals for topographic proportions were estimated using a nonparametric participant-level cluster bootstrap with 20,000 resamples. Participants were resampled by identifier, thereby preserving paired right- and left-sided observations within each bootstrap cluster. Percentile bootstrap confidence limits corresponding to the 2.5th and 97.5th percentiles were reported.

Circular descriptive statistics were additionally calculated for the sector positions. For each anatomical structure, the circular mean clock position and mean resultant length (R) were estimated overall and according to side. Values of R closer to 1 indicate greater concentration around the circular mean. Confidence intervals for the circular mean were estimated using the same participant-level cluster bootstrap procedure with 20,000 resamples.

Because right- and left-sided observations were obtained from the same participants, right–left comparisons of sector distributions were performed using paired Stuart–Maxwell tests. The Holm procedure was applied to adjust for multiple comparisons across the four anatomical structures.

Morphometric measurements were analyzed using linear mixed-effects models to account for the dependence of bilateral observations obtained from the same participant. Three principal mixed-effects models were constructed: one for the intercord distances (LC/MC, LC/PC, and MC/PC), one for the cord-to-axillary-artery distances (LC/AA, MC/AA, and PC/AA), and one for the cord-to-axillary-vein distances (LC/AV, MC/AV, and PC/AV).

In each model, distance type, side, sex, and their interactions were included as fixed effects, and participant identifier was included as a random intercept. Degrees of freedom for fixed-effect tests were estimated using the Satterthwaite approximation.

When a statistically significant overall effect of distance type was identified, estimated marginal means were used for model-based pairwise comparisons among the three distance types. Pairwise *p*-values were adjusted using the Holm procedure. Estimated marginal means were used for inference, whereas the primary descriptive morphometric values reported in the tables are the directly observed arithmetic means and standard deviations.

All statistical tests were two-sided, and statistical significance was set at *p* < 0.05.

## 3. Results

### 3.1. Participant Characteristics and Evaluability

Fifty participants were included in the study, comprising 25 women and 25 men. All participants underwent bilateral examination, yielding 100 potential side-specific infraclavicular examinations.

The mean participant age was 41.50 ± 12.72 years, mean height was 1.72 ± 0.10 m, mean body weight was 71.28 ± 13.01 kg, and mean BMI was 23.89 ± 3.03 kg/m^2^ (Table 1).

Values are presented as mean ± SD unless otherwise indicated.

The LC was adequately visualized for quantitative assessment in 94 of 100 examinations, the MC in 89, and the PC in 83. The AV was identified in all 100 examinations. Consequently, LC/MC measurements were available in 83 examinations, whereas LC/PC and MC/PC measurements were available in 77 examinations each.

### 3.2. Topographic Distribution of the Brachial Plexus Cords and Axillary Vein Relative to the Axillary Artery

**Figure 4:**
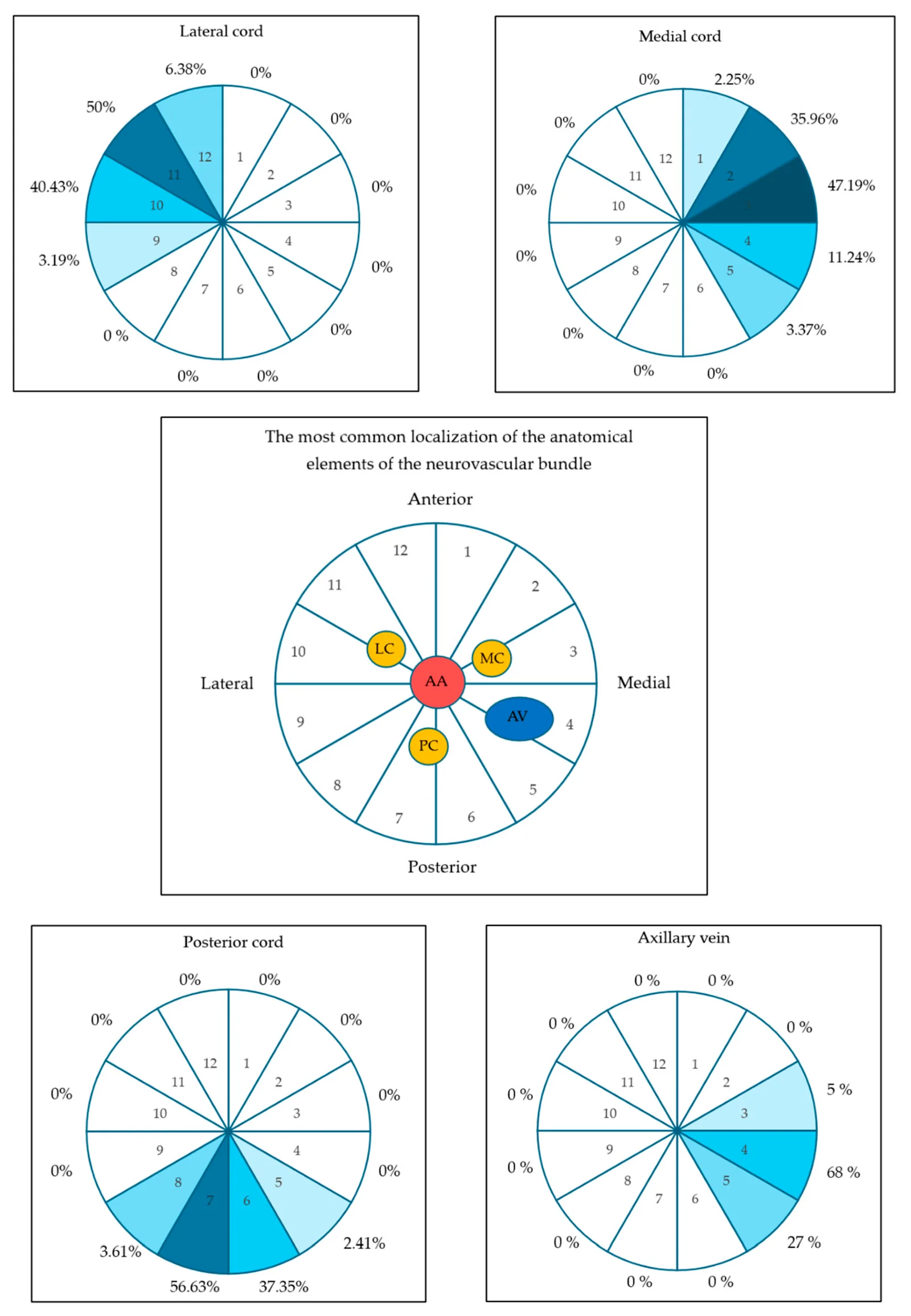
Topographic distribution of the Brachial Plexus Cords and Axillary Vein around the Axillary Artery and their most common localization around the AA.

The brachial plexus cords and AV demonstrated concentrated distributions around the AA (Table 2). The LC was located in sectors 10–11 in 85/94 examinations (90.4%; 95% CI, 84.4–95.7%), the MC in sectors 2–4 in 84/89 examinations (94.4%; 95% CI, 89.2–98.9%), the PC in sectors 6–7 in 78/83 examinations (94.0%; 95% CI, 88.6–98.8%), and the AV in sectors 4–5 in 95/100 examinations (95.0%; 95% CI, 90.0–99.0%). More specifically, the LC was most frequently located in sector 11 (47/94, 50.0%), followed by sector 10 (38/94, 40.4%), sector 12 (6/94, 6.4%), and sector 9 (3/94, 3.2%). The MC was most frequently located in sector 3 (42/89, 47.2%), followed by sector 2 (32/89, 36.0%), sector 4 (10/89, 11.2%), sector 5 (3/89, 3.4%), and sector 1 (2/89, 2.2%). The PC was most frequently located in sector 7 (47/83, 56.6%), followed by sector 6 (31/83, 37.3%), sector 8 (3/83, 3.6%), and sector 5 (2/83, 2.4%). The AV was most frequently located in sector 4 (68/100, 68.0%), followed by sector 5 (27/100, 27.0%) and sector 3 (5/100, 5.0%). The results are presented in Figure 4. Detailed right- and left-sided topographic distributions are provided in Table S1, while circular descriptive characteristics of the sector positions are presented in Table S2.

The 95% confidence intervals were estimated using a nonparametric participant-level cluster bootstrap with 20,000 resamples. LC, lateral cord; MC, medial cord; PC, posterior cord; AV, axillary vein.

Paired Stuart–Maxwell analyses did not demonstrate statistically significant right–left differences in sector distribution for the LC (*p* = 0.7728; Holm-adjusted *p* = 1.0000), MC (*p* = 0.3863; adjusted *p* = 1.0000), PC (*p* = 0.4953; adjusted *p* = 1.0000), or AV (*p* = 0.0907; adjusted *p* = 0.3629). Thus, the observed side-specific differences were interpreted as descriptive rather than evidence of systematic anatomical asymmetry. Detailed results of the paired Stuart–Maxwell analyses are provided in Table S3.

### 3.3. Morphometric Relationships

The directly observed morphometric measurements are summarized in Table 3.

#### 3.3.1. Intercord Distances

**Figure 5:**
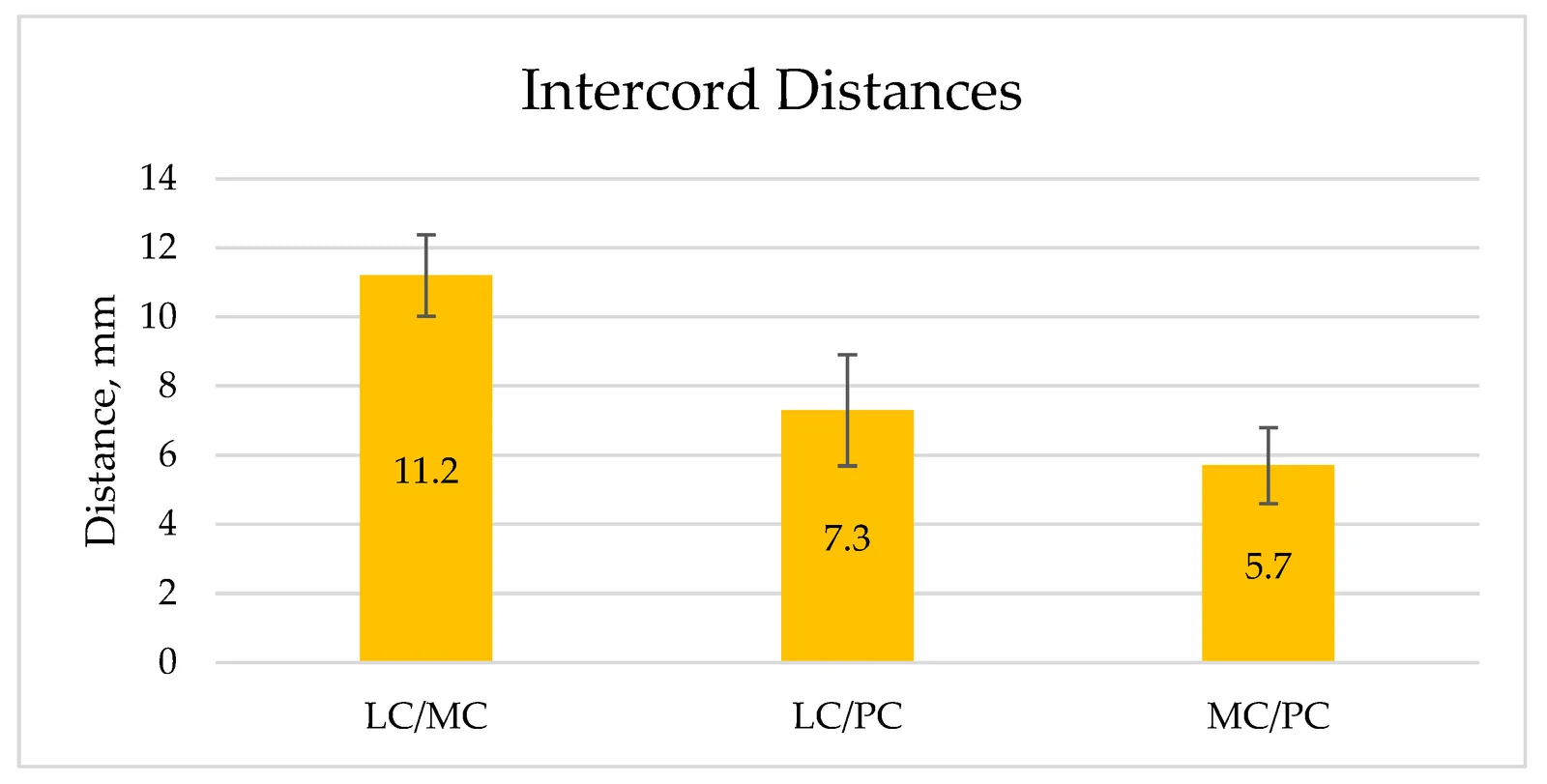
Intercord distances between the brachial plexus cords in the infraclavicular region. Values are presented as mean ± standard deviation.

The greatest observed mean intercord distance was LC/MC (11.20 ± 1.18 mm), followed by LC/PC (7.30 ± 1.61 mm), whereas MC/PC was the shortest (5.70 ± 1.10 mm) (Figure 5). Side- and sex-specific descriptive intercord measurements are provided in Table S4.

The mixed-effects analysis included 237 available measurements from 50 participants. A statistically significant main effect of distance type was observed, F(2, 182.9) = 438.996, *p* < 0.001. No significant overall effect of side was identified, F(1, 206.8) = 0.417, *p* = 0.519, and there was no significant overall effect of sex, F(1, 47.7) = 0.857, *p* = 0.359. The investigated interactions were not statistically significant.

Model-based pairwise comparisons demonstrated significant differences between all three intercord distances after Holm adjustment (all adjusted *p* < 0.001), confirming the relationship: MC/PC < LC/PC < LC/MC. Detailed mixed-effects model results, estimated marginal means, and pairwise comparisons for the intercord distances are provided in Table S5.

#### 3.3.2. Distances from the Cords to the Axillary Artery

**Figure 6:**
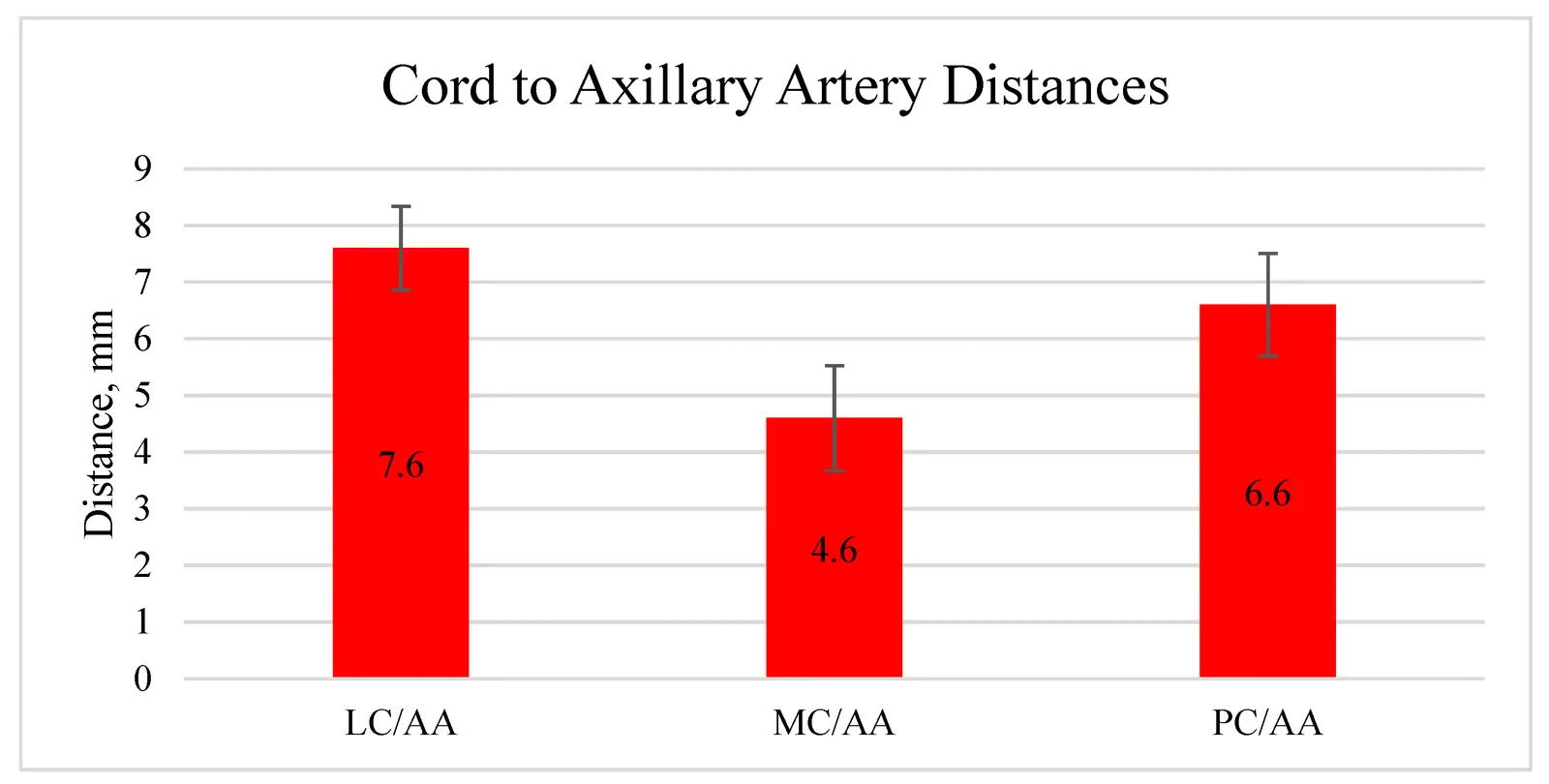
Distances from the lateral, medial, and posterior cords of the brachial plexus to the axillary artery. Values are presented as mean ± standard deviation.

The observed mean LC/AA distance was 7.60 ± 0.74 mm, the MC/AA distance was 4.60 ± 0.93 mm, and the PC/AA distance was 6.60 ± 0.91 mm (Figure 6).

The mixed-effects analysis included 266 available measurements from 50 participants. A significant main effect of distance type was identified, F(2, 212.4) = 323.514, *p* < 0.001. There was no significant overall effect of side, F(1, 216.0) = 0.153, *p* = 0.696, or sex, F(1, 49.2) = 0.992, *p* = 0.324. None of the investigated interactions were statistically significant.

All pairwise differences among the three cord-to-AA distances remained significant after Holm adjustment (all adjusted *p* < 0.001). The observed relationship was MC/AA < PC/AA < LC/AA. Detailed mixed-effects model results, estimated marginal means, and pairwise comparisons for the cord-to-AA distances are provided in Table S6.

#### 3.3.3. Distances from the Cords to the Axillary Vein

**Figure 7:**
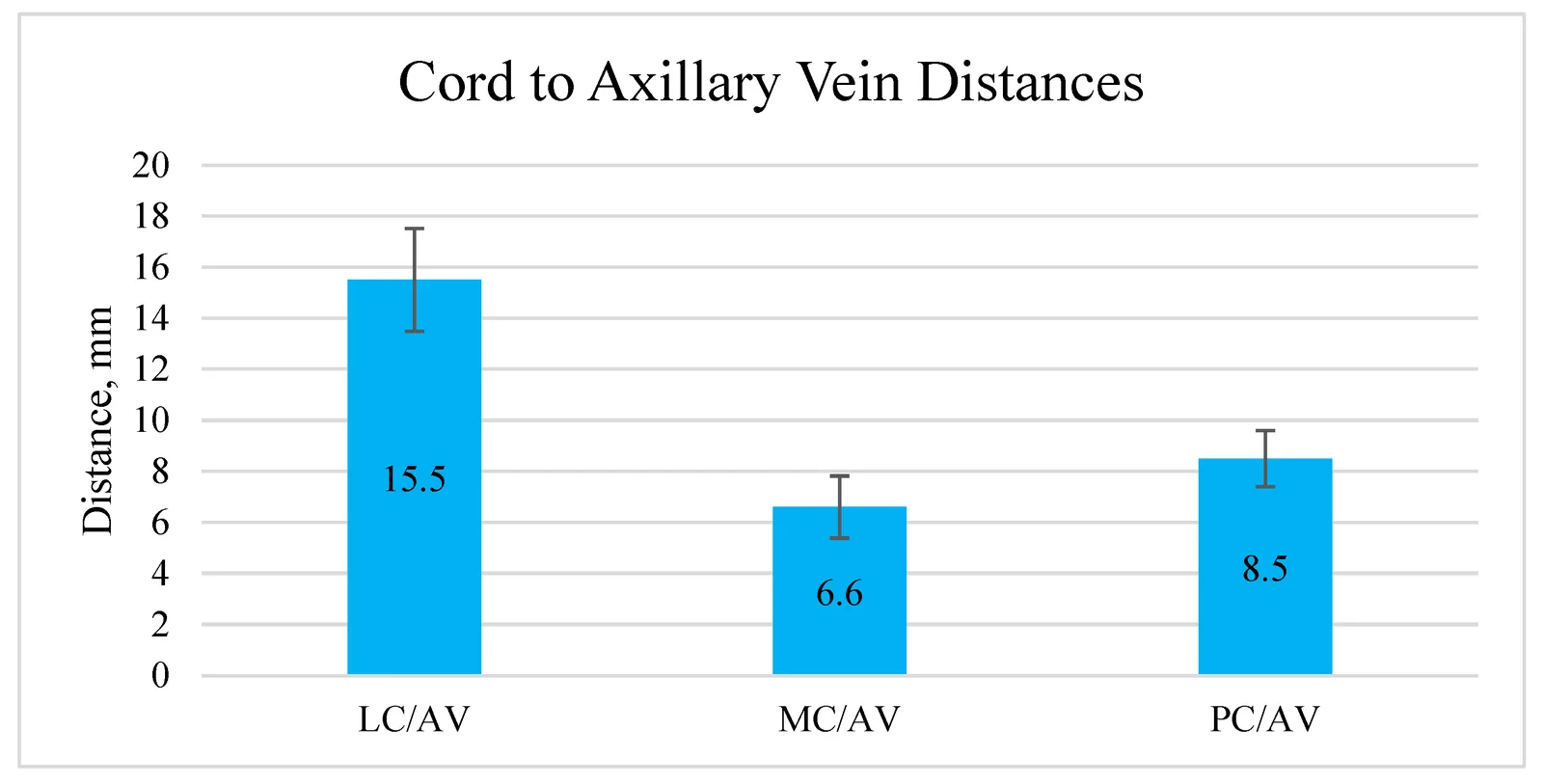
Distances from the lateral, medial, and posterior cords of the brachial plexus to the axillary vein. Values are presented as mean ± standard deviation.

The observed mean LC/AV distance was 15.50 ± 2.01 mm, the MC/AV distance was 6.60 ± 1.22 mm, and the PC/AV distance was 8.50 ± 1.10 mm (Figure 7).

The mixed-effects analysis included 266 available measurements from 50 participants. A significant main effect of distance type was identified, F(2, 209.2) = 1095.458, *p* < 0.001. No significant overall effect of side, F(1, 212.3) = 0.086, *p* = 0.770, or sex, F(1, 47.2) = 0.861, *p* = 0.358, was identified. None of the investigated interactions were statistically significant (Table 4).

## 4. Discussion

The present prospective multicenter study provides a bilateral ultrasonographic assessment of the topographic and morphometric relationships of the brachial plexus cords and axillary vessels in the infraclavicular region. The principal findings were a concentrated and consistent artery-centered topographic distribution, a clearly non-equidistant morphometric configuration of the three cords, and an absence of significant systematic right–left or sex-related effects in the principal analyses.

The present topographic findings closely correspond to the distribution reported by Kumar et al. [12], whose 12-sector artery-centered framework was adopted for the current analysis. Kumar et al. reported the MC in sectors 2–4 in 92% of observations, the PC in sectors 6–7 in 92%, the LC in sectors 10–11 in 89%, and the AV in sectors 4–5 in 95%. The corresponding proportions in the present study were 94.4%, 94.0%, 90.4%, and 95.0%, respectively. This agreement supports the consistency of the broad spatial pattern around the AA across studies, while the distribution across adjacent sectors emphasizes that the cords should not be regarded as occupying fixed clock-face positions.

The present study should be interpreted as a methodological replication and extension of the work by Kumar et al. rather than as an identical reproduction of their acquisition protocol. Kumar et al. examined the lateral infraclavicular fossa, whereas the present examinations were performed in the mid-infraclavicular region, approximately beneath the middle portion of the clavicle, using a parasagittal craniocaudal transducer orientation [12]. Despite this difference in acquisition plane, the broad artery-centered topographic distribution was closely reproduced. The present study additionally incorporated bilateral assessment and extended the morphometric analysis to intercord and individual cord-to-axillary-vein distances.

The availability of the individual cords for ultrasonographic assessment was broadly consistent with previous findings. Kumar et al. reported visualization rates of 90.5% for the LC, 87% for the MC, and 71% for the PC [12]. In the present bilateral dataset, the corresponding structure-specific availability was 94%, 89%, and 83%, respectively. In both studies, the PC was the least consistently visualized cord. Differences in ultrasound equipment, acquisition plane, tissue depth, individual anatomy, and operator-dependent factors may contribute to variation in neural conspicuity.

The present cord-to-AA measurements also permit comparison with previous sonographic data. Kumar et al. reported mean center-to-center distances of approximately 7.2 mm for the LC, 6.7 mm for the MC, and 6.2 mm for the PC [12]. In the present study, the observed means were 7.60 mm, 4.60 mm, and 6.60 mm, respectively. Both studies therefore identified the LC as relatively distant from the center of the AA, whereas the current dataset demonstrated a distinctly shorter MC/AA distance. These differences reinforce the importance of treating precise morphometric values as plane- and population-dependent rather than universal constants.

Previous MRI-based investigation has likewise demonstrated that the infraclavicular cords occupy a non-uniform spatial arrangement around the AA [13]. The present study extends this concept by quantifying the distances among the cords themselves. The LC/MC distance was greatest, LC/PC was intermediate, and MC/PC was shortest. These findings demonstrate that the three cords do not form an equidistant triangular arrangement within the examined sonographic plane.

The relationship between the cords and the AV was also markedly non-uniform. The MC demonstrated the shortest measured center-to-center relationship with the vein, followed by the PC, whereas the LC was substantially farther from the AV. Thus, within the imaging plane used in the present study, the MC had the shortest measured relationship with both major vascular structures.

The acquisition plane used in the present study should also be distinguished from the costoclavicular approach. Anatomical investigations of the costoclavicular space have demonstrated a more compact arrangement of the brachial plexus cords in that region [14]. A recent systematic review and meta-analysis likewise distinguished classical infraclavicular and costoclavicular approaches as anatomically and technically different ultrasound-guided approaches [15]. The present mid-infraclavicular parasagittal plane should therefore not be interpreted as a costoclavicular acquisition.

The AA remains an important sonographic reference structure in infraclavicular regional anesthesia, as demonstrated in early ultrasound-guided techniques [16]. Different needle and injection approaches have subsequently been compared in clinical studies [17]. Perivascular injection strategies, including the “double bubble” concept [18] and single- versus double-injection approaches [19], illustrate the clinical relevance of understanding the distribution of the cords around the vessel. Posterior parasagittal approaches have also been described, with sonographic and cadaveric evaluation of injectate distribution around the AA and brachial plexus [20].

However, the center-to-center measurements reported here should not be interpreted as direct procedural safety margins. The distance between the centers of a cord and vessel is different from the minimum edge-to-edge distance between their anatomical boundaries. Neural dimensions, vessel diameter, vessel compression, tissue deformation, transducer pressure, and needle trajectory would all affect the actual separation encountered during an intervention. Accordingly, these measurements provide quantitative anatomical imaging data rather than a recommended needle clearance distance.

Similarly, anatomical proximity should not be interpreted as evidence that a specific local anesthetic deposition strategy will reliably produce complete neural coverage. Tissue-plane barriers within the infraclavicular neurovascular region may influence injectate distribution. Cadaveric work by Brenner et al. demonstrated that local tissue planes can restrict dye spread [21]. Cadaveric comparisons of different infraclavicular approaches further demonstrate that the anatomy encountered during needle advancement depends on the selected trajectory and approach [22]. The present study did not investigate injectate spread or clinical block outcomes.

The bilateral design is an important extension of previous unilateral sonographic work. Paired analysis of the 12-sector distribution showed no significant systematic right–left difference for any of the four structures. Similarly, the mixed-effects morphometric models did not identify significant overall effects of side. These findings do not establish absolute bilateral anatomical equivalence but provide no evidence of a consistent directional asymmetry in this cohort.

No significant overall sex effect was identified for any of the three morphometric distance families. Because the sample included 25 women and 25 men, the study allowed balanced descriptive assessment by sex; nevertheless, the total number of independent participants remains limited, and smaller sex-related differences cannot be excluded.

Several limitations should be considered. First, the study included 50 independent participants. Although bilateral examination yielded up to 100 side-specific observations, these observations were correlated within participants and cannot be interpreted as an independent sample of 100 individuals. Participant-level cluster bootstrap procedures and linear mixed-effects analyses were therefore used where appropriate to account for the bilateral structure of the data. Arm circumference was not prospectively recorded and therefore could not be included in the anthropometric analyses.

Second, not every cord could be visualized sufficiently for every measurement. Rather than excluding the entire participant, the corresponding values were treated as structure-specific missing data. The PC had the highest proportion of missing observations, which may reflect its deeper location and more difficult sonographic visualization.

Third, ultrasonography is inherently operator- and acquisition-dependent. Transducer angle, anisotropy, imaging depth, body habitus, gain, focus, probe pressure, and the selected frozen frame may influence both neural conspicuity and apparent anatomical relationships. The AV is particularly susceptible to compression and deformation.

In addition, examinations were performed using different ultrasound platforms at the participating centers. Although the same general imaging and measurement protocol was applied, differences in transducer characteristics and system-specific image processing may have contributed to variability.

Fourth, all morphometric measurements used in the primary analysis were obtained by a single primary operator. Inter- and intra-observer reproducibility were not formally assessed; therefore, the reproducibility of anatomical identification, center-point placement, and morphometric measurements across observers remains to be established.

Fifth, all examinations were obtained with a standardized upper-limb position and in a defined mid-infraclavicular parasagittal plane. Alterations in shoulder position, arm abduction, transducer orientation, or acquisition level may modify the apparent relative positions of the cords and vessels.

Finally, the study was anatomical and ultrasonographic and did not evaluate needle trajectory, local anesthetic distribution, block onset, block success, volume requirements, vascular puncture, neurological complications, or other clinical outcomes. The clinical implications of the observed relationships should therefore be considered translational interpretations of the imaging anatomy rather than directly demonstrated procedural effects.

## 5. Conclusions

Bilateral ultrasonographic examination demonstrated a consistent topographic organization of the lateral, medial, and posterior cords of the brachial plexus and the AV around the AA in the mid-infraclavicular region. The observed sector distributions were closely aligned with the previously described 12-sector artery-centered framework.

Quantitative morphometry demonstrated a non-equidistant arrangement of the cords. The MC showed the shortest center-to-center distance to both the AA and AV, whereas the LC was the most distant from both vessels. No significant systematic right–left or sex-related effects were identified in the principal morphometric analyses.

These findings provide quantitative ultrasonographic reference data for infraclavicular neurovascular anatomy. Because the measurements were center-to-center and the study did not assess procedural outcomes, the reported distances should not be interpreted as direct needle safety margins or as predictors of block efficacy.

## Figures and Tables

**Table 1:** Participant characteristics.

Characteristic	Overall (N = 50)	Women (N = 25)	Men (N = 25)	Overall Range
Age, years	41.50 ± 12.72	41.68 ± 13.97	41.32 ± 11.64	20–60
Height, m	1.72 ± 0.10	1.71 ± 0.11	1.74 ± 0.10	1.56–1.90
Body weight, kg	71.28 ± 13.01	69.68 ± 14.09	72.88 ± 11.91	47–108
BMI, kg/m^2^	23.89 ± 3.03	23.66 ± 3.06	24.12 ± 3.05	17.96–32.00

**Table 2:** Topographic distribution of the brachial plexus cords and axillary vein around the axillary artery.

Structure	Sector	n/N	%	95% CI
LC	9	3/94	3.2	0.0–7.2
LC	10	38/94	40.4	34.4–45.7
LC	11	47/94	50.0	45.2–54.8
LC	12	6/94	6.4	2.1–11.6
MC	1	2/89	2.2	0.0–5.7
MC	2	32/89	36.0	28.7–43.0
MC	3	42/89	47.2	39.8–54.4
MC	4	10/89	11.2	5.4–18.1
MC	5	3/89	3.4	0.0–7.6
PC	5	2/83	2.4	0.0–6.1
PC	6	31/83	37.3	29.8–44.6
PC	7	47/83	56.6	48.8–64.6
PC	8	3/83	3.6	0.0–8.1
AV	3	5/100	5.0	1.0–10.0
AV	4	68/100	68.0	62.0–75.0
AV	5	27/100	27.0	20.0–34.0

**Table 3:** Observed morphometric measurements.

Measurement	N	Mean ± SD, mm	95% CI for the Mean, mm	Median (IQR)	Min–Max
**Intercord distances**					
LC/MC	83	11.20 ± 1.18	10.94–11.46	10.95 (1.19)	8.82–14.57
LC/PC	77	7.30 ± 1.61	6.94–7.67	7.25 (1.69)	4.41–11.41
MC/PC	77	5.70 ± 1.10	5.45–5.95	5.54 (1.59)	3.64–8.09
**Cord-to-axillary-artery distances**					
LC/AA	94	7.60 ± 0.74	7.45–7.75	7.71 (0.90)	5.84–9.39
MC/AA	89	4.60 ± 0.93	4.41–4.80	4.74 (0.95)	2.17–7.04
PC/AA	83	6.60 ± 0.91	6.40–6.80	6.60 (1.08)	4.21–8.51
**Cord-to-axillary-vein distances**					
LC/AV	94	15.50 ± 2.01	15.09–15.92	15.35 (1.89)	12.22–22.20
MC/AV	89	6.60 ± 1.22	6.34–6.86	6.26 (1.58)	4.80–10.04
PC/AV	83	8.50 ± 1.10	8.26–8.74	8.33 (1.45)	6.62–11.15

AA, axillary artery; AV, axillary vein; LC, lateral cord; MC, medial cord; PC, posterior cord. Values are presented as directly observed mean ± SD, 95% confidence interval for the mean, median (interquartile range), and minimum–maximum range, as applicable.

**Table 4:** Principal fixed effects from the three linear mixed-effects models.

Distance Family	Measurements	Distance Type	Side	Sex
Intercord	237	F(2,182.9) = 438.996; *p* < 0.001	F(1,206.8) = 0.417; *p* = 0.519	F(1,47.7) = 0.857; *p* = 0.359
Cord-to-AA	266	F(2,212.4) = 323.514; *p* < 0.001	F(1,216.0) = 0.153; *p* = 0.696	F(1,49.2) = 0.992; *p* = 0.324
Cord-to-AV	266	F(2,209.2) = 1095.458; *p* < 0.001	F(1,212.3) = 0.086; *p* = 0.770	F(1,47.2) = 0.861; *p* = 0.358

All investigated interactions were non-significant. All pairwise differences remained significant after Holm adjustment (all adjusted *p* < 0.001), demonstrating the relationship: MC/AV < PC/AV < LC/AV. Detailed mixed-effects model results, estimated marginal means, and pairwise comparisons for the cord-to-AV distances are provided in Table S7.

## Data Availability

The data presented in this study are available from the corresponding author upon reasonable request. The data are not publicly available due to privacy and ethical restrictions related to participant data.

## Supplementary Materials

The following supporting information can be downloaded at https://www.mdpi.com/article/10.3390/jcm15176892/s1, Table S1, Right- and left-sided topographic distribution of the brachial plexus cords and axillary vein; Table S2, Circular characteristics of the topographic positions overall and according to side; Table S3, Paired Stuart–Maxwell comparison of right- and left-sided sector distributions; Table S4, Descriptive intercord distances according to side and sex; Table S5, Detailed linear mixed-effects analysis of intercord distances; Table S6, Detailed linear mixed-effects analysis of cord-to-axillary-artery distances; Table S7, Detailed linear mixed-effects analysis of cord-to-axillary-vein distances.

## Abbreviations

The following abbreviations are used in this manuscript:

LC	Lateral Cord
MC	Medial Cord
PC	Posterior Cord
AA	Axillary Artery
AV	Axillary Vein
PMJM	Pectoralis Major Muscle
PMNM	Pectoralis Minor Muscle
