# Supplementary material for: Bilateral Ultrasonographic Mapping and Morphometry of the Infraclavicular Brachial Plexus: A Prospective Multicenter Study
Source: J Clin Med. 2026 Sep 6;15(17):6892. doi: 10.3390/jcm15176892 (PMC13565863; doi:10.3390/jcm15176892)
Supplement: Supplementary file 1 [file jcm-15-06892-s001.zip › jcm-4484268-supplementary.pdf]

Supplementary Materials

Table S1. Right- and left-sided topographic distribution of the brachial plexus cords and axillary vein

Table S1A. Individual sector distribution by side

Structure	Sector	Right, n/N (%) [95% CI]	Left, n/N (%) [95% CI]
LC	9	2/45 (4.4) [0.0–11.4]	1/49 (2.0) [0.0–6.2]
LC	10	17/45 (37.8) [23.9–52.3]	21/49 (42.9) [28.6–57.1]
LC	11	24/45 (53.3) [38.6–67.4]	23/49 (46.9) [32.7–61.2]
LC	12	2/45 (4.4) [0.0–11.4]	4/49 (8.2) [2.0–16.7]
MC	1	1/44 (2.3) [0.0–7.3]	1/45 (2.2) [0.0–7.0]
MC	2	19/44 (43.2) [28.9–58.1]	13/45 (28.9) [15.9–42.6]
MC	3	17/44 (38.6) [24.4–53.3]	25/45 (55.6) [40.9–70.2]
MC	4	5/44 (11.4) [2.3–21.4]	5/45 (11.1) [2.3–20.9]
MC	5	2/44 (4.5) [0.0–11.6]	1/45 (2.2) [0.0–7.0]
PC	5	1/43 (2.3) [0.0–7.5]	1/40 (2.5) [0.0–7.9]
PC	6	15/43 (34.9) [20.9–50.0]	16/40 (40.0) [25.0–55.3]
PC	7	26/43 (60.5) [45.5–75.0]	21/40 (52.5) [36.8–68.2]
PC	8	1/43 (2.3) [0.0–7.5]	2/40 (5.0) [0.0–12.8]
AV	3	1/50 (2.0) [0.0–6.0]	4/50 (8.0) [2.0–16.0]
AV	4	31/50 (62.0) [48.0–76.0]	37/50 (74.0) [62.0–86.0]
AV	5	18/50 (36.0) [22.0–50.0]	9/50 (18.0) [8.0–30.0]

Note: Confidence intervals were estimated using a nonparametric participant-level cluster bootstrap with 20,000 resamples. LC, lateral cord; MC, medial cord; PC, posterior cord; AV, axillary vein.

Table S1B. Dominant combined sector groups by side

Structure	Dominant sectors	Right, n/N (%) [95% CI]	Left, n/N (%) [95% CI]
LC	10–11	41/45 (91.1) [81.8–97.9]	44/49 (89.8) [80.0–98.0]
MC	2–4	41/44 (93.2) [84.8–100.0]	43/45 (95.6) [88.9–100.0]
PC	6–7	41/43 (95.3) [88.1–100.0]	37/40 (92.5) [83.3–100.0]
AV	4–5	49/50 (98.0) [94.0–100.0]	46/50 (92.0) [84.0–98.0]

Note: Confidence intervals were estimated using a nonparametric participant-level cluster bootstrap with 20,000 resamples.

Table S2. Circular characteristics of the topographic positions overall and according to side

Structure	Side	N	Mean clock position	95% CI	R
LC	Overall	94	10.60	10.47–10.72	0.942

MC	Overall	89	2.76	2.59–2.95	0.916
PC	Overall	83	6.62	6.50–6.74	0.952
AV	Overall	100	4.22	4.12–4.31	0.963
LC	Right	45	10.58	10.39–10.77	0.944
MC	Right	44	2.70	2.46–2.97	0.904
PC	Right	43	6.63	6.46–6.80	0.956
AV	Right	50	4.34	4.20–4.48	0.964
LC	Left	49	10.61	10.42–10.80	0.941
MC	Left	45	2.82	2.60–3.03	0.928
PC	Left	40	6.60	6.41–6.80	0.948
AV	Left	50	4.10	3.96–4.24	0.966

Note: R denotes the mean resultant length, with values closer to 1 indicating greater circular concentration. Confidence intervals for the circular mean were estimated using participant-level cluster bootstrap resampling.

Table S3. Paired Stuart–Maxwell comparison of right- and left-sided sector distributions

Structure	Paired N	χ^2	df	p	Holm-adjusted p
LC	44	1.118	3	0.7728	1.0000
MC	39	4.148	4	0.3863	1.0000
PC	33	2.391	3	0.4953	1.0000
AV	50	4.800	2	0.0907	0.3629

Note: Only participants with valid observations on both sides for the corresponding structure were included. Holm adjustment was performed across the four anatomical structures using the unrounded original p-values.

Table S4. Descriptive intercord distances according to side and sex

Table S4A. Descriptive intercord distances according to side

Measurement	Side	N	Mean ± SD, mm	Median (IQR), mm	Min–Max, mm
LC/MC	Right	39	11.03 ± 1.09	10.94 (1.10)	9.12–14.57
LC/MC	Left	44	11.35 ± 1.25	10.98 (1.38)	8.82–14.19
LC/PC	Right	38	7.33 ± 1.66	7.23 (1.79)	4.41–11.03
LC/PC	Left	39	7.28 ± 1.59	7.31 (1.57)	4.61–11.41
MC/PC	Right	39	5.62 ± 1.01	5.44 (1.46)	3.91–7.72
MC/PC	Left	38	5.79 ± 1.19	5.67 (2.00)	3.64–8.09

Table S4B. Descriptive intercord distances according to sex

Measurement	Sex	N	Mean ± SD, mm	Median (IQR), mm	Min–Max, mm
LC/MC	Women	40	11.21 ± 1.39	10.83 (1.29)	8.82–14.57
LC/MC	Men	43	11.20 ± 0.96	11.01 (1.07)	9.87–13.92

LC/PC	Women	39	7.41 ± 1.33	7.41 (1.50)	4.41–9.81
LC/PC	Men	38	7.19 ± 1.87	6.86 (2.20)	4.61–11.41
MC/PC	Women	39	5.90 ± 1.16	5.80 (1.85)	3.76–8.09
MC/PC	Men	38	5.50 ± 1.01	5.44 (1.25)	3.64–7.65

Note: Descriptive values correspond to the available structure-specific observations.

Table S5. Detailed linear mixed-effects analysis of intercord distances

Table S5A. Detailed linear mixed-effects analysis of intercord distances: omnibus fixed-effect tests

Effect	F	df	df(res)	p
Distance type	438.996	2	182.9	<0.0001
Side	0.417	1	206.8	0.5192
Sex	0.857	1	47.7	0.3592
Distance type × Side	0.662	2	182.8	0.5171
Distance type × Sex	0.438	2	182.9	0.6458
Side × Sex	0.437	1	206.8	0.5095
Distance type × Side × Sex	0.008	2	182.8	0.9924

Table S5B. Detailed linear mixed-effects analysis of intercord distances: estimated marginal means

Measurement	EMM, mm	SE	95% CI
LC/MC	11.21	0.155	10.91–11.52
LC/PC	7.33	0.160	7.01–7.64
MC/PC	5.71	0.159	5.40–6.03

Table S5C. Detailed linear mixed-effects analysis of intercord distances: Holm-adjusted pairwise comparisons

Comparison	Estimated difference, mm	Holm-adjusted p
LC/MC – LC/PC	+3.89	<0.0001
LC/MC – MC/PC	+5.50	<0.0001
LC/PC – MC/PC	+1.61	<0.0001

Note: The model included 237 available measurements from 50 participants. Distance type, side, sex, and their interactions were included as fixed effects, with participant identifier as a random intercept. Satterthwaite degrees of freedom were used.

Table S6. Detailed linear mixed-effects analysis of cord-to-axillary-artery distances

Table S6A. Detailed linear mixed-effects analysis of cord-to-axillary-artery distances: omnibus fixed-effect tests

Effect	F	df	df(res)	p

Distance type	323.514	2	212.4	<0.0001
Side	0.153	1	216.0	0.6959
Sex	0.992	1	49.2	0.3241
Distance type × Side	0.075	2	212.7	0.9279
Distance type × Sex	1.229	2	212.4	0.2948
Side × Sex	0.404	1	216.0	0.5259
Distance type × Side × Sex	0.387	2	212.7	0.6795

Table S6B. Detailed linear mixed-effects analysis of cord-to-axillary-artery distances: estimated marginal means

Measurement	EMM, mm	SE	95% CI
LC/AA	7.60	0.095	7.41–7.79
MC/AA	4.61	0.097	4.42–4.81
PC/AA	6.60	0.100	6.40–6.80

Table S6C. Detailed linear mixed-effects analysis of cord-to-axillary-artery distances: Holm-adjusted pairwise comparisons

Comparison	Estimated difference, mm	Holm-adjusted p
LC/AA – MC/AA	+2.99	<0.0001
LC/AA – PC/AA	+1.00	<0.0001
MC/AA – PC/AA	−1.99	<0.0001

Note: The model included 266 available measurements from 50 participants. Distance type, side, sex, and their interactions were included as fixed effects, with participant identifier as a random intercept. Satterthwaite degrees of freedom were used.

Table S7. Detailed linear mixed-effects analysis of cord-to-axillary-vein distances

Table S7A. Detailed linear mixed-effects analysis of cord-to-axillary-vein distances: omnibus fixed-effect tests

Effect	F	df	df(res)	p
Distance type	1095.458	2	209.2	<0.0001
Side	0.086	1	212.3	0.7697
Sex	0.861	1	47.2	0.3583
Distance type × Side	0.151	2	209.5	0.8598
Distance type × Sex	1.238	2	209.2	0.2920
Side × Sex	0.026	1	212.3	0.8721
Distance type × Side × Sex	0.269	2	209.5	0.7648

Table S7B. Detailed linear mixed-effects analysis of cord-to-axillary-vein distances: estimated marginal means

Measurement	EMM, mm	SE	95% CI
LC/AV	15.53	0.174	15.18–15.87
MC/AV	6.61	0.177	6.26–6.96
PC/AV	8.48	0.182	8.12–8.84

Table S7C. Detailed linear mixed-effects analysis of cord-to-axillary-vein distances: Holm-adjusted pairwise comparisons

Comparison	Estimated difference, mm	Holm-adjusted p
LC/AV – MC/AV	+8.92	<0.0001
LC/AV – PC/AV	+7.04	<0.0001
MC/AV – PC/AV	−1.87	<0.0001

Note: The model included 266 available measurements from 50 participants. Distance type, side, sex, and their interactions were included as fixed effects, with participant identifier as a random intercept. Satterthwaite degrees of freedom were used.
